# Neutrophil-to-lymphocyte ratio as a promising non-invasive biomarker for symptom assessment and diagnosis of interstitial cystitis/bladder pain syndrome

**DOI:** 10.1186/s12894-023-01353-z

**Published:** 2023-11-08

**Authors:** Hanwei Ke, Lin Zhu, Qi Wang, Kexin Xu

**Affiliations:** 1 Department of Urology, Peking University People’s Hospital, Beijing, 100044 China; 2 Peking University Applied Lithotripsy Institute, Peking University People’s Hospital, Beijing, 10034 China; 3 Department of Plastic Surgery, Affiliated Beijing Chaoyang Hospital of Capital Medical University, Beijing, China

**Keywords:** Biomarker, Diagnosis, Interstitial cystitis, Neutrophil-to-lymphocyte ratio, Urodynamic Disease

## Abstract

**Background:**

Our study aims to investigate the association between the serum neutrophil-to-lymphocyte ratio (NLR) and interstitial cystitis (IC), as well as to explore whether NLR can serve as a diagnostic marker to distinguish IC from overactive bladder (OAB). We postulate that elevated NLR levels are intricately linked to the onset and clinical presentation of IC, and that the NLR profiles in OAB patients exhibit discernible disparities from those of IC patients.

**Methods:**

In a retrospective analysis, we scrutinized the medical records of 70 women diagnosed with IC/BPS, 20 women diagnosed with OAB, and a randomly selected cohort of 150 healthy women who underwent physical examinations during the same temporal frame. A comprehensive panel of blood tests was administered to all participants, and NLR was determined through the calculation of the neutrophil-to-lymphocyte proportion. Additionally, symptom assessment questionnaires and urination diaries were collected from IC/BPS patients.

**Results:**

NLR levels exhibited significant distinctions among the IC/BPS, Normal, and OAB groups (P < 0.001). Within the IC/BPS group, Hunner type interstitial cystitis (HIC) demonstrated notably divergent NLR levels in comparison to non-Hunner type interstitial cystitis (NHIC) (p = 0.001). Additionally, we observed positive correlations between NLR and Nighttime voids (r = 0.268, p = 0.029), ICPI (r = 0.327, p = 0.007), ICSI (r = 0.369, p = 0.002), PUF Symptom Scale (r = 0.263, p = 0.032), and PUF (r = 0.297, p = 0.015). The receiver operating characteristic (ROC) analysis yielded an area under the curve (AUC) of 0.765 for NLR in distinguishing IC/BPS from the Normal group, and an AUC of 0.707 in discerning IC from OAB. Furthermore, the AUC of NLR was 0.723 for identifying HIC and NHIC patients.

**Conclusions:**

Our study unveils the prospective utility of serum NLR as a promising biomarker for both diagnostic and symptom evaluation purposes in IC/BPS patients. It effectively demarcates this condition from OAB, which presents with similar clinical features. Consequently, NLR demonstrates potential as a non-invasive diagnostic instrument to distinguish between the subtypes of IC, particularly HIC and NHIC, which manifest similar symptoms within the IC/BPS spectrum.

**Supplementary Information:**

The online version contains supplementary material available at 10.1186/s12894-023-01353-z.

## Introduction

Interstitial cystitis/bladder pain syndrome (IC/BPS) is an enduring inflammatory ailment of the urinary bladder, distinguished by the presence of discomfort or pelvic pain upon bladder fullness, in tandem with lower urinary tract symptoms, including heightened frequency and urgency [1]. Given the dearth of definitive objective indicators for IC/BPS diagnosis, the recognition of this condition primarily relies on symptomatic evaluation. Unfortunately, the lack of uniform diagnostic criteria for IC/BPS has given rise to significant heterogeneity in the outcomes of epidemiological investigations surrounding this ailment.

IC/BPS encompasses distinct subtypes, including both ulcerative and non-ulcerative variations, each potentially underpinned by differing pathophysiological mechanisms and clinical attributes [2, 3]. The diagnosis of IC/BPS hinges upon the presence of persistent pelvic pain, pressure, or discomfort deemed to be associated with the bladder, in conjunction with a minimum of one additional urinary symptom, such as heightened urinary frequency or urgency. The categorization of BPS is informed by observations from cystoscopy and the morphological characteristics elucidated through bladder biopsy [4].At present, the identification of IC/BPS is primarily contingent upon the symptomatic profile presented by the patient and necessitates subsequent validation through invasive and costly procedures, namely cystoscopy and bladder biopsy [5]. Cystoscopic scrutiny has unveiled that the majority of IC/BPS cases exhibit glomerulations within the bladder, with only a modest 5%~15% exhibiting Hunner’s ulcers [6]. However, it is imperative to acknowledge that glomerulation can also manifest in other bladder disorders, thereby diminishing its specificity. Such manifestations may occur in patients afflicted with bacterial cystitis, radiation cystitis, and even in those with an otherwise healthy bladder [7]. Interstitial cystitis/bladder pain syndrome and overactive bladder (OAB) represent clinical syndromes primarily delineated by the symptomatic narratives presented by patients. It is generally presumed that IC/BPS and OAB can be differentiated on the basis of patient-reported symptoms. For instance, urge incontinence is regarded as a rarity in IC/BPS, while bladder pain is seldom attributed to OAB. However, the linkage of urgency and urgency incontinence exclusively to OAB, and the association of frequency/nocturia and bladder pain solely with IC/BPS, are not always founded on a rational basis. Clinical investigations have furnished compelling evidence suggesting the potential for overlap between these two conditions [8]. Pain is conventionally acknowledged as a distinctive hallmark of IC/BPS, while urgency and urge incontinence are the defining characteristics of OAB [9]. Nevertheless, studies have demonstrated that this differentiation is not an unequivocal demarcation but rather emblematic of a continuum within the sphere of bladder hypersensitivity syndrome [10]. Recent research has accentuated the presence of inflammatory biomarkers in both biopsied bladder tissues and urine specimens from patients grappling with overactive bladder (OAB), even in the absence of urinary tract infections [11, 12]. The process of diagnosis and the selection of appropriate therapeutic interventions are often complicated by the overlapping symptomatology witnessed in patients grappling with IC/BPS and OAB. Furthermore, a persistent inflammatory disposition emerges as a prevailing shared attribute among patients afflicting with both IC/BPS and OAB. Hence, reliance solely on clinical symptoms is an unreliable means to differentiate IC/BPS from OAB, occasionally necessitating invasive procedures such as cystoscopy or bladder biopsy to procure ailment-specific data for the patient. To circumvent the burdens of cost and the associated perils linked to invasive examinations, the development of non-invasive markers for IC/BPS diagnosis is imperative. Typically, the diagnosis and treatment of maladies are intricate processes that may not be achieved at an early stage, thereby underscoring the pressing demand for an objective and pertinent IC/BPS screening biomarker.

Two decades ago, Zahorec and colleagues introduced a novel parameter for gauging immune-inflammatory responses and neuro-endocrine stress, which has since been christened as the neutrophil-to-lymphocyte ratio (NLR) [13]. NLR is derived by dividing the neutrophil count by the lymphocyte count. Previous research has documented NLR as an inflammatory marker with associations to chronic maladies [14], The NLR is acknowledged as a straightforward metric for evaluating an individual’s inflammatory condition [13], Furthermore, NLR has exhibited diagnostic accuracy in foreseeing sepsis and infectious pathologies [15], Ischemic stroke [16], cancer [17], Major cardiac events [18] in patients. This readily accessible objective metric can be ascertained through a standard blood examination, rendering it a cost-effective and straightforward calculation. The integration of NLR into the diagnostic regimen and its applicability in monitoring immune-inflammatory reactions to diverse provocations can substantially assist healthcare practitioners in formulating precise diagnoses and well-informed judgments. With a well-established two-decade legacy of NLR deployment as a prognostic gauge, ample substantiation endorses its consistent integration within clinical practice. The NLR parameter holds the potential to serve as an invaluable screening tool, offering early predictive indicators, stratifying diseases according to their severity, and forecasting prognostic outcomes, thereby elevating the quality of patient care.

The objective of this study is to investigate the relationship between NLR and IC/BPS, and whether NLR can be used to distinguish IC/BPS from OAB.

## Materials & methods

This cross-sectional investigation retrospectively examined the medical records of 70 adult females diagnosed with interstitial cystitis and 20 adult females diagnosed with overactive bladder at Peking University People’s Hospital, spanning from January 2012 to October 2022. Additionally, data were obtained from a randomly selected cohort of 150 healthy women who underwent physical examinations during the same timeframe. Ethical approval for this study was obtained from the Medical Ethics Committee of Peking University People’s Hospital (approval number: 2022PHB400-001). Inclusion criteria for patients with interstitial cystitis mandated compliance with the diagnostic criteria established by the American Urological Association. These criteria necessitated the presence of unpleasant sensations such as pain, pressure, or discomfort in the urinary bladder, coupled with lower urinary tract symptoms enduring for more than 6 weeks, while ruling out any discernible infections or underlying causes [19]. Similarly, inclusion criteria for patients with overactive bladder necessitated adherence to the established diagnostic criteria. These criteria encompassed the presence of urgency, with or without urge urinary incontinence, typically accompanied by heightened daytime frequency and nocturia [20]. Furthermore, participants undergoing cancer treatment, those with potential bladder organ lesions affecting bladder function, individuals with other severe medical conditions, and those identified with acute urinary tract infections through urinalysis were excluded from the study. All participants underwent standard blood, biochemical, and urine examinations, with examinations avoiding the menstrual period.

The Body Mass Index (BMI) was computed by dividing the body weight in kilograms by the square of height in meters. Blood samples were procured following a fasting period of more than 10 h. Standard tests encompassed the assessment of white blood cell count (WBC), absolute lymphocyte count (LYMPH), absolute neutrophil count (NEUT), uric acid (UA), serum creatinine (Scr), hypertension (HP), diabetes (DM), proteinuria, urine pH (UPH), and serum globulin (SG). The Neutrophil-to-Lymphocyte Ratio (NLR) was derived by dividing the neutrophil count by the lymphocyte count. Previous medical history was scrutinized to ascertain whether patients had a history of high blood pressure and diabetes. Additional data collected from IC/BPS patients included the duration of illness, voiding diary records (daytime voids, nighttime voids, total urination, average voided volumes), Visual Analogue Scale/Score (VAS), Interstitial Cystitis Problem Index (ICPI), Interstitial Cystitis Symptom Index (ICSI), Pelvic Pain and Urgency/Frequency questionnaire (PUF), PUF Symptom Scale, PUF Distress Scale, and overactive bladder syndrome score (OABSS). The presence of Hunner’s ulcers was ascertained during cystoscopy conducted with hydrodistention.

Baseline characteristics analysis stratified participants into three groups: one group comprised individuals diagnosed with IC/BPS, another included those who underwent physical examinations during the same temporal span, and the third encompassed individuals diagnosed with OAB. Continuous variables adhering to a normal distribution were presented as mean ± standard deviation (SD), while non-normally distributed variables were portrayed as median (IQR, interquartile range). Data following a normal distribution were represented as percentages. The Student’s t-test was employed for normally distributed continuous data, while the Wilcoxon rank-sum test was utilized for non-normally distributed data. Spearman correlation analysis was applied to assess the relationship between rating scales and IC/BPS. ROC analysis was conducted using the ‘pROC’ package in R (version 4.2.1) to ascertain the optimal cutoff value. Subsequently, the results were visualized using the ‘ggplot2’ package (version 3.3.6).

## Results

LYMPH levels (1.61 ± 0.55 vs. 2.13 ± 1.73) in the IC/BPS group were notably lower than those in the normal group. Conversely, NLR levels (2.59 ± 1.28 vs. 1.64 ± 0.5), NEYT (3.74 ± 1.26 vs. 3.17 ± 0.95), age (51.23 ± 16.72 vs. 43.28 ± 6.92), and BMI (23.8 ± 3.78 vs. 21.95 ± 2.92) were distinctly higher in the IC/BPS group. Moreover, patients with IC/BPS exhibited elevated body weight, WBC, Scr, and SG levels, along with diminished height, UA, and UPH levels compared to the normal group. It is worth noting that these differences did not reach statistical significance. When comparing the IC/BPS group to the OAB group, most parameters showed no statistically significant disparities, except for NLR and age. Specifically, the IC/BPS group demonstrated a significantly higher NLR (2.59 ± 1.28) compared to both the OAB (1.77 ± 0.47) and normal (1.64 ± 0.5) groups. However, there was no statistically significant difference in NLR between the OAB and normal groups (Table 1).

**Table 1:** Baseline characteristics for each group

Characteristic	Normal	IC/BPS	OAB	P value	IC/BPS vs. Normal	Normal vs. OAB	IC/BPS vs. OAB
n	150	70	20				
HP, n (%)				0.311	1.000	0.218	0.265
0	107 (44.6%)	50 (20.8%)	11 (4.6%)				
1	43 (17.9%)	20 (8.3%)	9 (3.8%)				
DM, n (%)				0.333	0.809	0.235	0.454
0	136 (56.7%)	62 (25.8%)	16 (6.7%)				
1	14 (5.8%)	8 (3.3%)	4 (1.7%)				
Proteinuria, n (%)				0.071	0.594	0.068	0.213
0	148 (61.7%)	68 (28.3%)	18 (7.5%)				
1	2 (0.8%)	2 (0.8%)	2 (0.8%)				
NLR	1.64 ± 0.5	2.59 ± 1.28	1.77 ± 0.47	< 0.001	< 0.001	0.247	< 0.001
Age, year	43.28 ± 6.92	51.23 ± 16.72	64.4 ± 10.76	< 0.001	< 0.001	< 0.001	< 0.001
Height, cm	162.07 ± 4.51	161.04 ± 6.15	158.7 ± 6.4	0.018	0.163	0.003	0.140
Weight, Kg	57.65 ± 7.97	61.71 ± 10.22	62.48 ± 12.74	0.010	0.004	0.114	0.782
BMI	21.95 ± 2.92	23.8 ± 3.78	24.65 ± 3.9	< 0.001	< 0.001	0.007	0.381
WBC, 109/L	5.62 ± 1.32	6.03 ± 1.72	5.75 ± 1.83	0.175	0.057	0.702	0.531
LYMPH#, 109/L	2.13 ± 1.73	1.61 ± 0.55	1.79 ± 0.49	0.037	0.016	0.387	0.197
NEUT#, 109/L	3.17 ± 0.95	3.74 ± 1.26	3.12 ± 1.08	< 0.001	< 0.001	0.834	0.047
Scr umol/L	59.35 ± 13.42	60.21 ± 11.04	63.55 ± 24.02	0.690	0.638	0.452	0.553
UA umol/L	291.92 ± 70.12	300.87 ± 78.51	303.5 ± 71.07	0.611	0.397	0.489	0.893
UPH	6.04 ± 0.83	6.03 ± 0.62	5.92 ± 0.71	0.819	0.909	0.556	0.316
SG	1.02 ± 0.01	1.02 ± 0.01	1.01 ± 0.01	0.102	0.632	0.025	0.062

Data are presented as mean ± SD, n (%), median (IQR). Values were calculated using a t-test and χ2 test. BMI, Body mass index; WBC, White blood cell count; LYMPH, Lymphocyte count; NEUT, Neutrophil count; NLR, Neutrophil-to-lymphocyte ratio; UA, uric acid; Scr, Serum creatinine; HP, Hypertension; DM, Diabetes Mellitus; UPH, Urine pH; SG, Urine specific gravity; IC, Interstitial cystitis. * P < 0.05

To minimize the potential impact of confounding factors, such as age, height, weight, and BMI, between the IC/BPS group and the control group, we employed propensity score matching (PSM) to harmonize the two cohorts based on these variables, resulting in a final cohort of 38 matched pairs. As illustrated in Table 2, a notable discrepancy in NLR (2.48 ± 1.18 vs. 1.62 ± 0.492) was evident between the IC/BPS group and the control group, suggesting the potential of NLR as a discriminative marker for distinguishing individuals with IC from their healthy counterparts. In a multivariate regression analysis comparing the IC/BPS group to the normal group, the regression coefficient for NLR was determined to be 0.241, with a p-value less than 0.001. The model’s F-value was 6.299, and the adjusted R-squared (R²) was 0.266 (Table S1).

**Table 2:** Baseline characteristics for IC/BPS and Normal group after propensity score matching

	Normal	IC/BPS	P-value
n	38	38	
NLR	1.62 ± 0.492	2.48 ± 1.18	< 0.001
Age, year	46.2 ± 8.33	45.1 ± 12.5	0.652
Height, cm	162 ± 4.70	161 ± 5.69	0.498
Weight, Kg	59.2 ± 11.3	59.8 ± 9.53	0.806
BMI	22.5 ± 4.24	22.9 ± 3.09	0.632
WBC, 109/L	5.66 ± 1.06	5.93 ± 1.32	0.342
LYMPH, 109/L	2.03 ± 0.564	1.63 ± 0.526	0.00242
NEUT, 109/L	3.10 ± 0.724	3.69 ± 1.14	0.00854
Scr, umol/L	59.2 ± 8.91	59.8 ± 11.3	0.796
UA, umol/L	299 ± 68.9	286 ± 64.0	0.423
DM	0.105 ± 0.311	0.0526 ± 0.226	0.402
Proteinuria	0 ± 0	0.0263 ± 0.162	0.324
UPH	6.03 ± 0.771	6.04 ± 0.619	0.935
SG	1.02 ± 0.00669	1.02 ± 0.00913	0.468
HP	0.289 ± 0.460	0.158 ± 0.370	0.173

Data are presented as mean ± SD, n (%), median (IQR). Values were calculated using a t-test and χ2 test. BMI, Body mass index; WBC, White blood cell count; LYMPH, Lymphocyte count; NEUT, Neutrophil count; NLR, Neutrophil-to-lymphocyte ratio; UA, uric acid; Scr, Serum creatinine; HP, Hypertension; DM, Diabetes Mellitus; UPH, Urine pH; SG, Urine specific gravity; IC, Interstitial cystitis. * P < 0.05

We categorized IC/BPS patients into two subtypes, namely, Hunner type interstitial cystitis (HIC) and non-Hunner type interstitial cystitis (NHIC), based on the presence or absence of Hunner ulcers during cystoscopy with hydrodistention. We observed significant variations in NLR between patients with HIC (3.34 ± 1.66) and NHIC (2.14 ± 0.71). However, there were no statistically significant disparities in urinary diary parameters, disease duration, VAS, ICPI, ICSI, PUF Symptom Scale, PUF Distress Scale, or OABSS (Table 3). These findings suggest that NLR may serve as a valuable tool for distinguishing between different subtypes of IC/BPS, which traditional symptom scales may be unable to differentiate.

**Table 3:** The rating scales and urodynamic data of HIC and NHIC

Characteristic	NHIC	HIC	p
n	44	26	
NLR	2.14 ± 0.71	3.34 ± 1.66	0.001
Medical history, months	57.52 ± 60.71	76.46 ± 119.36	0.39
Daytime voids, ml	20.19 ± 10.71	17.5 ± 9.41	0.308
Nighttime voids, ml	6.51 ± 3.7	7.46 ± 3.61	0.315
Total voids, ml	26.7 ± 11.82	23.04 ± 12.77	0.231
Average voided volumes, ml	160.06 ± 106.95	140 ± 69.66	0.44
VAS	5.07 ± 2.94	6.17 ± 2.81	0.142
ICPI	11.49 ± 2.81	12.83 ± 3.37	0.085
ICSI	11.86 ± 3.89	12.92 ± 4.94	0.338
PUF Symptom Scale	12.93 ± 3.79	14.29 ± 4.85	0.207
PUF distress Scale	7.07 ± 2.86	7.62 ± 2.76	0.443
PUF	20 ± 5.91	21.92 ± 6.91	0.235
OABSS	7.49 ± 2.41	7.5 ± 3.22	0.987

Data are presented as mean ± SD, n (%), median (IQR). NLR, Neutrophil-to-lymphocyte ratio; VAS, Visual Analogue Scale/Score; ICSI, Interstitial Cystitis Symptom Index; ICPI, Interstitial Cystitis Problem Index; PUF, Pelvic Pain and Urgency/Frequency questionnaire; OABSS, overactive bladder syndrome score

**Fig. 1:**
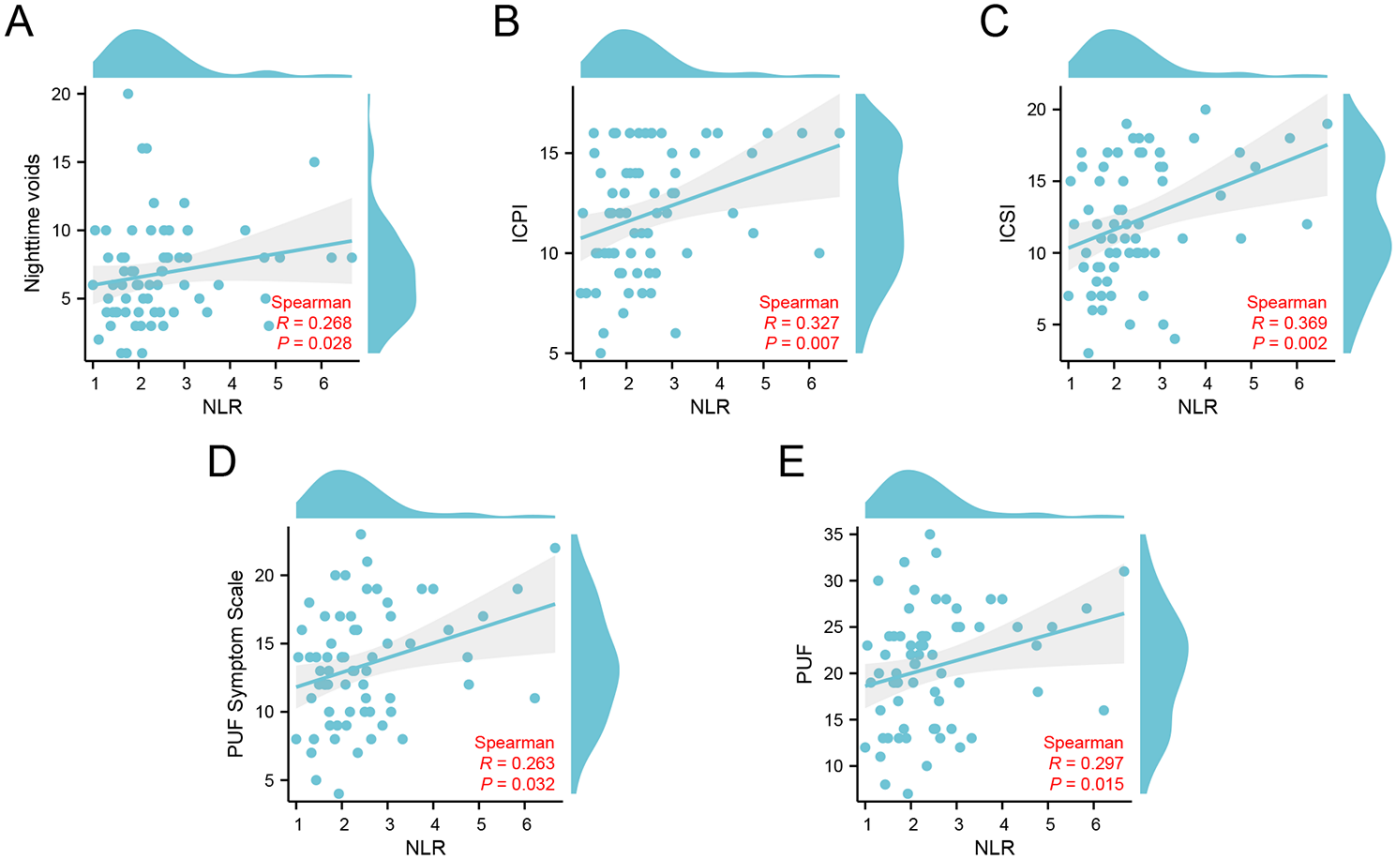
Correlation scatterplot of NLR with IC symptom score. **(A)** Correlation scatterplot of NLR and ICPI, **(B)** Correlation scatterplot of NLR and ICSI.

To investigate the relationship between symptoms and NLR in patients with interstitial cystitis, we conducted Spearman correlation analysis to assess the connection between NLR and various scoring scales and urinary diaries (Table 4). Our analysis revealed a significant positive correlation between NLR and Nighttime voids (r = 0.268, p = 0.029), ICPI (r = 0.327, p = 0.007), ICSI (r = 0.369, p = 0.002), PUF Symptom Scale (r = 0.263, p = 0.032), and PUF (r = 0.297, p = 0.015) (Fig. 1).

**Table 4:** Correlation coefficients between rating scales and urodynamic data with NLR in patients with IC/BPS

Characteristic	Overall	Spearson	P value
Medical history, months	64.76 ± 87.51	-0.111	0.366
Daytime voids, ml	19.22 ± 10.27	-0.06	0.63
Nighttime voids, ml	6.85 ± 3.67	0.268*	0.028
Total voids, ml	25.32 ± 12.22	-0.048	0.696
Average voided volumes, ml	152.18 ± 93.88	-0.018	0.898
VAS	5.46 ± 2.92	0.111	0.369
ICPI	11.97 ± 3.06	0.327**	0.007
ICSI	12.24 ± 4.29	0.369**	0.002
PUF Symptom Scale	13.42 ± 4.21	0.263*	0.032
PUF distress Scale	7.27 ± 2.82	0.205	0.096
PUF	20.69 ± 6.3	0.297*	0.015
OABSS	7.49 ± 2.7	0.184	0.137

Data are presented as mean ± SD, n (%), median (IQR). NLR, Neutrophil-to-lymphocyte ratio; VAS, Visual Analogue Scale/Score; ICSI, Interstitial Cystitis Symptom Index; ICPI, Interstitial Cystitis Problem Index; PUF, Pelvic Pain and Urgency/Frequency questionnaire; OABSS, overactive bladder syndrome score

**Fig. 2:**
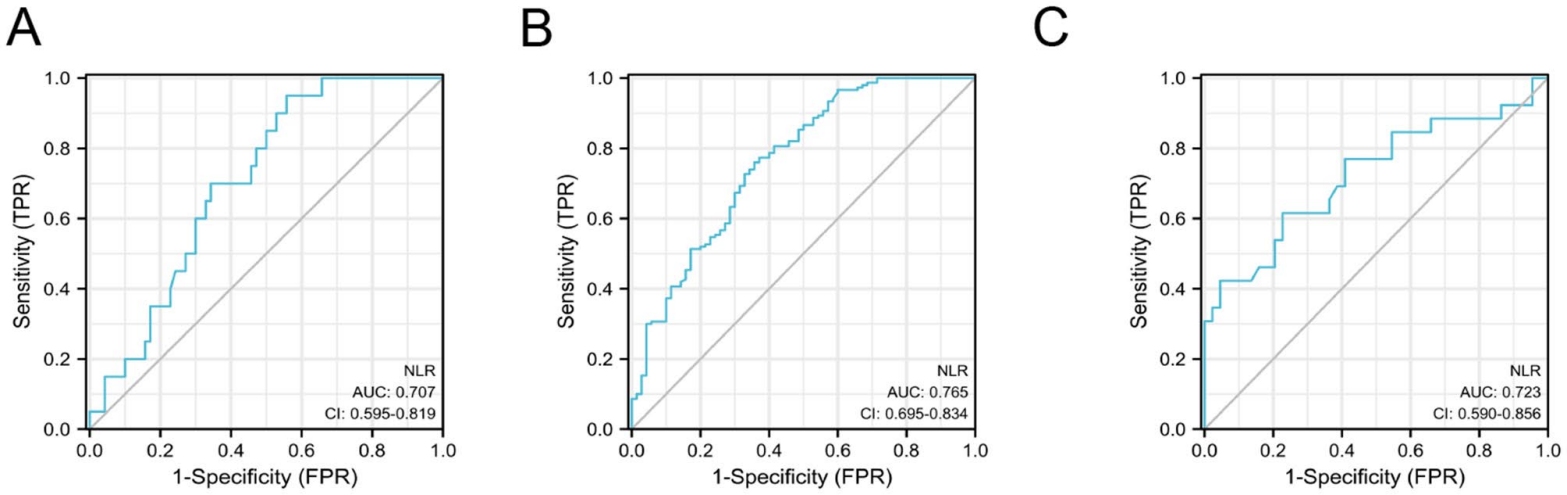
Receiver operating characteristic (ROC) curve of NLR. **(A)** NLR predicted ROC of IC/BPS group and OAB group, **(B)** NLR predicted ROC between IC/BPS group and Normal group, **(C)** NLR predicted the ROC of HIC group and NHIC group

Table 5 provides a summary of the diagnostic performance of the neutrophil-to-lymphocyte ratio (NLR) in distinguishing patients with IC/BPS from normal controls, IC/BPS from OAB, and HIC from NHIC. The AUC for NLR was 0.765 when discriminating between normal and IC/BPS groups, and the AUC was 0.707 when distinguishing between OAB and IC/BPS groups, indicating a high level of diagnostic accuracy. Furthermore, the AUC for NLR was 0.723 when identifying patients with HIC and NHIC, signifying a valuable diagnostic tool for distinguishing between these two subtypes of IC/BPS (Fig. 2). These results underscore the potential of NLR as a diagnostic biomarker for IC and as a means of excluding other conditions.

**Table 5:** Diagnostic values of NLR in patients with IC (from Normal controls and OAB)

	AUC	Cut-off value	Sensiticity	Specificity	PPV	NPV	Youden index
IC/BPS vs. OAB	0.707	2.397	0.95	0.443	0.328	0.969	0.393
IC/BPS vs. Normal	0.765	1.958	0.76	0.643	0.82	0.556	0.403
HIC vs. NHIC	0.723	2.543	0.615	0.773	0.615	0.773	0.388

## Discussion

Our study revealed that women with IC/BPS exhibited a significantly elevated neutrophil-to-lymphocyte ratio (NLR) compared to healthy individuals, and this distinction remained significant when compared to patients with overactive bladder (OAB). Through ROC analysis, we established that NLR possesses substantial diagnostic utility in discriminating between IC/BPS and OAB, as well as in distinguishing IC/BPS patients from those without the condition. Additionally, we identified a positive and statistically significant correlation between NLR and the symptom scales ICPI and ICSI, suggesting the potential of NLR as a symptom marker in IC/BPS patients. These findings imply that NLR holds promise as a marker for assessing symptoms in individuals with interstitial cystitis and offers a non-invasive diagnostic approach for detecting IC/BPS.

In previous investigations, potential biomarkers for IC/BPS have encompassed NGF, chemokines, inflammatory mediators, and particular antiproliferative agents. Within the urethra, NGF emanates from the smooth muscle of the bladder and the urothelium [21]. Jacobs et al. [22], discerned heightened urinary NGF levels in individuals with neurogenic bladder and central bladder symptoms. Women grappling with bladder dysfunction, encompassing idiopathic sensory urgency, chronic cystitis, and IC/BPS, manifested increased bladder NGF immunoreactivity in comparison to their counterparts without distressing symptoms, which included women with stress urinary incontinence [23]. Nevertheless, it’s imperative to note that urinary NGF levels also surge in cases of urinary tract infections, bladder outlet obstruction, and urinary calculi [24]. The usage of NGF as a biomarker for IC/BPS is hindered by its lack of specificity, making it inadequate for distinguishing these conditions from IC/BPS. Furthermore, quantifying NGF necessitates a more intricate technique, ELISA.

Chemokines exert their effects by interacting with seven-transmembrane G protein-coupled receptors situated on glycosaminoglycans tethered to the endothelial cell layer [25]. These chemokines are not limited to inducing chemotaxis but also have the capacity to activate target cells within the bladder, thereby contributing to the inflammation-driven alterations seen in IC/BPS. In a cross-sectional study, the hypothesis that urinary levels of specific chemokines are elevated in IC/BPS patients was tested. The findings revealed that urinary levels of chemokines/cytokines were 10–100 times higher in patients with both Hunner-type interstitial cystitis (HIC) and non-Hunner-type interstitial cystitis (NHIC) in comparison to asymptomatic controls [26]. JIANG et al. [27] observed that serum Scr levels, NGF, and pro-inflammatory cytokines/chemokines, such as IL-1β, IL-6, TNF-α, and IL-8, were significantly higher in IC/BPS patients than in the control group. The heightened levels of these pro-inflammatory cytokines (IL-1β, IL-6, TNF-α) and chemokines (IL-8) in the serum of IC/BPS patients not only suggest mast cell activation but also underline the significance of other inflammatory mediators in the pathogenesis of IC/BPS. Nevertheless, the application of inflammatory factors for diagnosing IC/BPS lacks specificity and is not suitable for widespread use.

A particular antiproliferative factor (AFP) has been identified in the urine of IC/BPS patients. AFP is the most extensively investigated biomarker and exhibits remarkable specificity and sensitivity [28]. Nevertheless, there is currently no evidence supporting the utilization of AFP for early detection of IC/BPS.

Neutrophils play a pivotal role in the innate immune response, carrying out essential functions like phagocytosis and releasing a multitude of cytokines and molecular mediators. The presence of lymphocytopenia is a hallmark of stress, while inflammation stems from processes like demargination, redistribution, and accelerated apoptosis. The neutrophil-to-lymphocyte ratio (NLR) serves as a valuable metric that reflects the equilibrium between the innate and adaptive immune responses. Consequently, NLR emerges as an exceptional indicator of both inflammation and stress, effectively capturing the intricate interplay between the two [29]. The underlying mechanism driving neutrophilia involves the activation of stem cell growth factor, which exerts its influence on neutrophil production [13]. In the early phases of an inflammatory response, the tumor necrosis factor family triggers apoptosis in lymphocytes, thus establishing lymphocytopenia as a diagnostic marker of infection [30]. Neutrophils possess the ability to release reactive oxygen species and an array of peptides, including antimicrobial peptides. These components contribute to the formation of neutrophil extracellular traps, which, unfortunately, exert adverse effects on the bladder [31]. In a comprehensive epidemiological study conducted by Lee et al. in 2018, a robust cohort comprising 12,160 healthy Korean citizens (6,268 men with a median age of 47 years and 5,892 women with a median age of 46 years) was meticulously examined. The researchers analyzed 12,160 blood samples that had undergone routine complete blood count assessments. The mean NLR value was (1.65 ± 0.79) [32]. Zahorec proposed NLR categorizations as follows: Latent, subclinical, or mild inflammatory/stress conditions: NLR range 2.3-3.0; Mild to moderate inflammation: NLR range 3–7; Moderate to severe inflammation, systemic infection, sepsis, and SIRS: NLR range 7–11; Severe inflammation, infection, severe sepsis and SIRS, and bacteremia: NLR range 11–17 [29]. In simpler terms, elevated NLR values are directly associated with conditions characterized by severe inflammation, stress, injury, trauma, major surgery, or cancer. These elevated NLR values indicate a worsening prognosis, signifying a higher likelihood of unfavorable outcomes in terms of illness or death.

NLR, as an objective measurement, can be readily derived from standard blood tests commonly conducted in clinical settings. Furthermore, in comparison to currently employed biomarkers, NLR presents itself as a cost-effective, easily obtainable, and readily calculable parameter. Therefore, introducing NLR into clinical practice is a feasible prospect. Our study suggests that NLR holds the potential to serve as a non-invasive diagnostic tool and a symptom indicator for interstitial cystitis, effectively distinguishing it from overactive bladder.

This study has a few limitations that warrant discussion. Firstly, the study’s cross-sectional nature restricts the establishment of causality between IC/BPS and NLR elevation, as the order of their occurrence cannot be ascertained. Future research adopting a prospective design and conducting multiple NLR measurements could yield more substantial evidence regarding NLR’s role in diagnosing IC/BPS. Secondly, it is conceivable that unmeasured covariates might have confounded the relationship between elevated NLR and IC/BPS. However, as the seemingly healthy individuals were selected from the general population, this potential confounding effect appears to have been mitigated. Thirdly, there is a possibility of bias, as various factors can influence the results of a routine blood test. Nevertheless, our random selection approach may have minimized some of the chance-related effects when screening the healthy control groups.

This study possesses several noteworthy advantages. Firstly, it stands as the inaugural examination of the correlation between NLR and IC/BPS. Secondly, our study utilized validated and widely accepted tools, namely the Interstitial Cystitis Symptom Index and Interstitial Cystitis Problem Index, to assess the IC status, enhancing the robustness and reliability of our findings.

## Conclusion

In summary, our study underscores the link between heightened NLR and the symptomatology and onset of IC/BPS, underscoring the potential of NLR as a diagnostic marker for IC/BPS. Moreover, NLR can function as an objective gauge of symptoms in IC/BPS patients. Nevertheless, the present study is constrained by sample size and the limited follow-up duration of IC/BPS patients. Subsequent investigations with more extensive sample sizes and protracted follow-up intervals are imperative to establish NLR as a dependable diagnostic marker for IC/BPS. Given the ongoing diagnostic debates surrounding IC/BPS, NLR can offer valuable support in the diagnostic process.

### Electronic supplementary material

Below is the link to the electronic supplementary material.

Supplementary Material 1

## Data Availability

The datasets used and/or analysed during the current study are available from the corresponding author on reasonable request.

## List of abbreviations

BMI: Body mass index
DM: Diabetes Mellitus
HP: Hypertension
IC: Interstitial cystitis
LYMPH#: Lymphocyte count/absolute concentration
NEUT#: Neutrophil count/absolute concentration
NLR: Neutrophil-to-lymphocyte ratio
OAB: Overactive bladder
Scr: Serum creatinine
SG: Urine specific gravity
UA: Uric acid
UPH: Urine pH
WBC: White blood cell count
