# Supplementary material for: Neutrophil-to-lymphocyte ratio as a promising non-invasive biomarker for symptom assessment and diagnosis of interstitial cystitis/bladder pain syndrome
Source: BMC Urol. 2023 Nov 8;23:180. doi: 10.1186/s12894-023-01353-z (PMC10633971; doi:10.1186/s12894-023-01353-z)
Supplement: Supplementary file 1 — Supplementary Material 1 [file 12894_2023_1353_MOESM1_ESM.docx]

Table S1, the multivariate regression analysis model for the IC/BPS group and normal group

|  | Regression Coefficient | Standardized Regression Coefficient | t-statistic | P value | F-statistic | Adjusted R^2^ |
| --- | --- | --- | --- | --- | --- | --- |
| NLR | 0.241 | 0.487 | 4.621 | <0.001 | 6.299 | 0.266 |
| Age, year | 0.01 | 0.246 | 3.411 | 0.001 |  |  |
| Height, cm | -0.009 | -0.094 | -0.25 | 0.803 |  |  |
| Weight, Kg | 0.009 | 0.175 | 0.199 | 0.842 |  |  |
| BMI | -0.006 | -0.044 | -0.052 | 0.959 |  |  |
| WBC, 109/L | 0.036 | 0.115 | 0.778 | 0.437 |  |  |
| LYMPH, 109/L | 0.003 | 0.008 | 0.115 | 0.909 |  |  |
| NEUT, 109/L | -0.074 | -0.173 | -0.952 | 0.342 |  |  |
| Scr umol/L | -0.003 | -0.07 | -0.946 | 0.345 |  |  |
| UA umol/L | 0 | 0.068 | 0.938 | 0.349 |  |  |
| HP, n (%) | -0.082 | -0.08 | -1.223 | 0.223 |  |  |
| DM, n (%) | -0.105 | -0.067 | -1.021 | 0.309 |  |  |
| Proteinuria, n (%) | 0.425 | 0.122 | 1.943 | 0.053 |  |  |
| UPH | 0.01 | 0.016 | 0.259 | 0.796 |  |  |
| SG | 4.73 | 0.077 | 1.238 | 0.217 |  |  |

BMI, Body mass index; WBC, White blood cell count; LYMPH, Lymphocyte count; NEUT, Neutrophil count; NLR, Neutrophil-to-lymphocyte ratio; UA, uric acid; Scr, Serum creatinine; HP, Hypertension; DM, Diabetes Mellitus; UPH, Urine pH; SG, Urine specific gravity; IC, Interstitial cystitis.
